# Dr Vaithianathan Satkunanayagam MBBS (Cey), DPM (Lond), FRCPsych (UK)

**DOI:** 10.1192/pb.bp.114.046854

**Published:** 2014-04

**Authors:** Anula Nikapota

**Figure:**
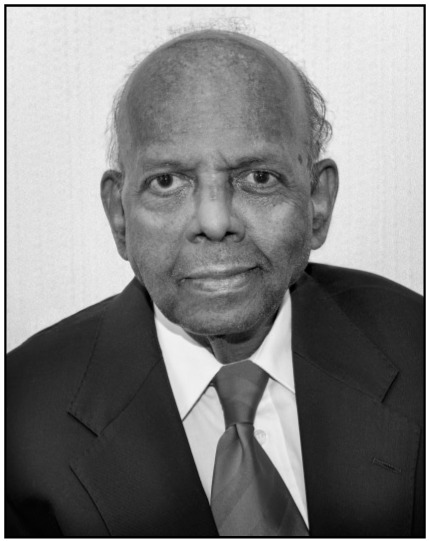


## Formerly Consultant Psychiatrist in Learning Disabilities, Manor and St Ebba’s hospitals, Surrey Heartlands NHS Trust

Dr Satkunanayagam, who died recently at the age of 87, was a psychiatrist who spanned two worlds in his professional life. He had many professional interests and areas of expertise, particularly with regard to alcohol and substance misuse. He was involved in postgraduate training both in Sri Lanka and the UK, and was an examiner for the MD Psychiatry (Sri Lanka) and MRCPsych (UK). However, his most significant contribution to mental health and well-being were his efforts to bring reconciliation and unity between divided ethnic groups.

Satkunam, as he was known among friends and colleagues in Sri Lanka, worked at a time when the pressures arising from ethnic tensions and then the civil war in Sri Lanka caused tension, bitterness and personal upheavals among many. He was one who tried unceasingly to create reconciliation, was never bitter and worked actively in this regard, while also working to help people within the Tamil community in the UK. In addition to being an active member of the Standing Committee of Tamil Speaking People (SCOT) and the Medical Institute of Tamils (MIOT) UK, along with myself and other mental health professionals - Sinhalese, Tamil and native British - he was a founder member of the UK Sri Lanka Trauma Group. The last conversation I had with him was about forming an association of Sinhalese and Tamils.

He was concerned that most Sri Lankan organisations in the UK were polarised with regard to ethnicity. His own life course was changed by the ethnic tensions which led him to leave his position as a senior psychiatrist in Colombo, Sri Lanka, and emigrate to the UK in 1981. He worked at Manor and St Ebba’s hospitals as a consultant psychiatrist in learning disabilities from 1981 until 1995.

Satkunam was born in May 1926 and educated in Jaffna, Northern Sri Lanka, and qualified in medicine in Colombo. He obtained his MRCPsych in 1963, completing postgraduate study at the Institute of Psychiatry, University of London, and returned to Sri Lanka to work in Colombo at the two major mental hospitals there, at Angoda and Mulleriyawa.

In his last few years he was partially disabled due to a stroke he suffered several years before. Nevertheless, he remained active both professionally and within charitable organisations. He went thrice to Lourdes on pilgrimages for people with special needs, the last only a month before his death from pneumonia.

Satkunam was a gentle man of great personal integrity and honesty. He loved people and their company and could surprise with his quiet but at times wicked sense of humour. He will be greatly missed by family, friends and colleagues in both countries.

I first met Satkunam when I returned to Sri Lanka to work as a child psychiatrist at the University of Colombo in 1978. He was a senior colleague who helped me a great deal to adjust to work and life in Sri Lanka.

Satkunam, who died on 6 October 2013, leaves his widow Rasu, his son Kuhan, daughter-in-law Christine and grandson Theo.

